# 2-Amino-4,6-dimethyl­pyrimidin-1-ium 2,3,5-triiodo­benzoate 2,3,5-triiodo­benzoic acid monosolvate

**DOI:** 10.1107/S160053681104534X

**Published:** 2011-11-09

**Authors:** Sevaiyan Malathy, Packianathan Thomas Muthiah

**Affiliations:** a School of Chemistry, Bharathidasan University, Tiruchirappalli 620 024, Tamilnadu, India

## Abstract

In the crystal structure of the title compound, C_6_H_10_N_3_^+^·C_7_H_2_I_3_O_2_^−^·C_7_H_3_I_3_O_2_, two *R*_2_^2^(8) motifs are observed. One is generated by the inter­action of the 2-amino-4,6-dimethyl­pyrimidin-1-ium cation with the carboxyl­ate group of the 2,3,5-triiodo­benzoate anion *via* N—H⋯O hydrogen bonds. The other *R*_2_^2^(8) motif is formed by the inter­action of two centrosymmentrically related pyrimidine moieties through N—H⋯N hydrogen bonds. The two motifs combine to form a linear heterotetra­meric unit. Heterotetra­meric units are linked by a carbox­yl–carboxyl­ate O—H⋯O hydrogen bond (involving the O—H group of neutral 2,3,5-triiodo­benzoic acid and an O atom of the anion), forming a supra­molecular chain along the *a* axis. In addition, components are held by weak I⋯O interactions in the range 3.023 (5) to 3.382 (5) Å and I⋯I inter­actions in the range 3.6327 (7) to 4.0025 (8) Å.

## Related literature

For the role of amino­pyrimidine– carboxyl­ate inter­actions see: Hunt *et al.* (1980 ▶); Baker & Santi (1965 ▶). For hydrogen-bond motifs, see: Bernstein *et al.* (1995 ▶); Etter (1990 ▶). For carbox­yl–carboxyl­ate inter­actions, see: Sawyer & James (1982 ▶). For iodine–iodine inter­actions, see: Stenzel *et al.* (1995 ▶). For halogen–oxygen inter­actions, see: Thalladi *et al.* (1996 ▶). For related structures see: Devi & Muthiah (2007 ▶); Ebenezer & Muthiah (2010 ▶).
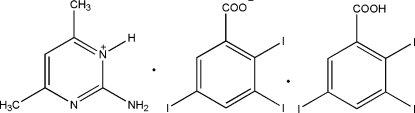


## Experimental

### 

#### Crystal data

C_6_H_10_N_3_^+^·C_7_H_2_I_3_O_2_^−^·C_7_H_3_I_3_O_2_
*M* *_r_* = 1122.75
Monoclinic, 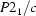

*a* = 9.4654 (2) Å
*b* = 9.6683 (2) Å
*c* = 31.1553 (5) Å
β = 90.366 (1)°
*V* = 2851.10 (10) Å^3^
*Z* = 4
Mo *K*α radiation
μ = 6.57 mm^−1^
*T* = 296 K
0.08 × 0.06 × 0.05 mm

#### Data collection

Bruker SMART APEXII CCD area-detector diffractometer
Absorption correction: multi-scan (*SADABS*; Bruker, 2008 ▶) *T*_min_ = 0.622, *T*_max_ = 0.735
34656 measured reflections
9270 independent reflections
6369 reflections with *I* > 2σ(*I*)
*R*_int_ = 0.032

#### Refinement

*R*[*F* ^2^ > 2σ(*F* ^2^)] = 0.051
*wR*(*F* ^2^) = 0.110
*S* = 1.03
9270 reflections
301 parameters
H-atom parameters constrained
Δρ_max_ = 3.16 e Å^−3^
Δρ_min_ = −2.46 e Å^−3^

### 

Data collection: *APEX2* (Bruker, 2008 ▶); cell refinement: *SAINT* (Bruker, 2008 ▶); data reduction: *SAINT*; program(s) used to solve structure: *SHELXS97* (Sheldrick, 2008 ▶); program(s) used to refine structure: *SHELXL97* (Sheldrick, 2008 ▶); molecular graphics: *PLATON* (Spek, 2009 ▶) and *POV-RAY* (Cason, 2004 ▶); software used to prepare material for publication: *PLATON*.

## Supplementary Material

Crystal structure: contains datablock(s) global, I. DOI: 10.1107/S160053681104534X/hg5115sup1.cif

Structure factors: contains datablock(s) I. DOI: 10.1107/S160053681104534X/hg5115Isup2.hkl

Supplementary material file. DOI: 10.1107/S160053681104534X/hg5115Isup3.cml

Additional supplementary materials:  crystallographic information; 3D view; checkCIF report

## Figures and Tables

**Table 1:** Hydrogen-bond geometry (Å, °)

*D*—H⋯*A*	*D*—H	H⋯*A*	*D*⋯*A*	*D*—H⋯*A*
N1—H1⋯O1*A*	0.86	1.80	2.652 (7)	170
N2—H2*B*⋯O2*A*	0.86	1.98	2.819 (8)	166
N2—H2*A*⋯N3^i^	0.86	2.19	3.042 (9)	172
O2*B*—H2⋯O2*A*^ii^	0.82	1.69	2.501 (7)	167

Symmetry codes: (i) 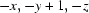; (ii) 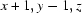.

**Fig. 1.**
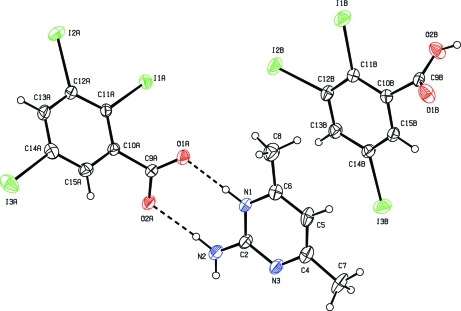
The asymmetric unit of (I), showing 30% probability displacement ellipsoids. Dashed lines indicate hydrogen bonds.

**Fig. 2.**
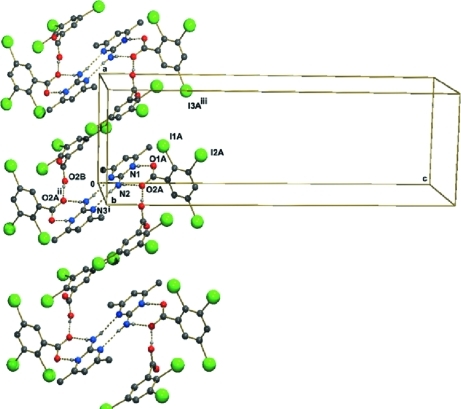
A view of supramolecular chain running along a axis. [symmetry codes: (i)-x,1 - y,-z; (ii) 1 + x,-1 + y,z; (iii) 1 + x, y, z]

## supplementary crystallographic information

### Comment

Aminopyrimidine derivatives are used as antifolate drugs. (Hunt *et al.*, 1980; Baker & Santi, 1965). 2,3,5-triiodobenzoic acid is an auxin polar transport inhibitor. Abnormal development of embryos induced by inhibitor results in plantlets without shoots and roots. The crystal structures of 2-amino-4,6-dimethyl pyrimidine terephthalic acid (Devi *et al.*, 2007) and 2-amino-4,6-dimethyl pyrimidine anthranilic acid (Ebenezer *et al.*, 2010) have been reported from our laboratory.

The asymmetric unit of the title compound (I) (Scheme.1) contains one 2-amino-4,6-dimethylpyrimidin-1-ium cation, one 2,3,5-triiodobenzoate anion and a neutral molecule of 2,3,5-triiodobenzoic acid as shown in Fig. 1.

Protonation at N1 position of the pyrimidine base is reflected by an increase in internal bond angle (C2—N1—C6= 121.8 (5) °) when compared with the unprotonated N3 atom of pyrimidine ring (C2—N3—C4=118.0 (6) °). The carboxylate group of the triiodobenzoate anion interacts with the protonated N1 and the 2-amino group of the pyrimidine moiety through a pair of N—H···O hydrogen bonds (Table. 1) to form robust *R*_2_^2^(8) ring motif (Etter, 1990; Bernstein *et al.*, 1995). In addition, another type of *R*_2_^2^(8) motif is formed by centrosymmetrically related pyrimidine molecules through a pair of N—H···N hydrogen bonds. These two different *R*_2_^2^(8) motifs generate a linear heterotetrameric unit (Ebenezer *et al.*, 2010).

The linear heterotetrameric units are interlinked through carboxyl- carboxylate interaction (Sawyer & James, 1982) *via* O—H···O hydrogen bond (involving carboxylic O—H group of neutral triiodobenzoic acid and carboxylate O atom of anion) and weak intermolecular I···O interaction. The presence of halogen-oxygen interaction is widely used in crystal engineering (Thalladi *et al.*,1996). These intermolecular interactions generate supramolecular chain along *a* axis. The crystal structure of (I) also exhibits weak intermolecular I···I interactions (Stenzel *et al.*,1995). The presence of weak intermolecular (I2A···I3A^iii^) (symmetry code: 1 + *x*, *y*, *z*) interaction links the supramolecular chain and generate supramolecular ladder like arrangement (Fig. 2).

### Experimental

Hot ethanolic solution of 2-amino-4,6-dimethylpyrimidine(30 mg, Aldrich) and 2,3,5-triiodobenzoic acid (125 mg, Loba Chemie) were mixed and warmed over a water bath for half an hour. The resulting solution was allowed to cool slowly at room temperature. After a week, brown coloured prismatic crystals were obtained.

### Refinement

All hydrogen atoms were positioned geometrically and were refined using a riding model. The N—H, O—H and C—H bond lengths are 0.86,0.82 and 0.93–0.96 Å, respectively [*U*_iso_(H)=1.2 *U*_eq_ (parent atom)].

### Crystal data

**Table:**

C_6_H_10_N_3_^+^·C_7_H_2_I_3_O_2_^−^·C_7_H_3_I_3_O_2_	*F*(000) = 2024
*M**_r_* = 1122.75	*D*_x_ = 2.616 Mg m^−^^3^
Monoclinic, *P*2_1_/*c*	Mo *K*α radiation, λ = 0.71073 Å
Hall symbol: -P 2ybc	Cell parameters from 9270 reflections
*a* = 9.4654 (2) Å	θ = 2.2–31.5°
*b* = 9.6683 (2) Å	µ = 6.57 mm^−^^1^
*c* = 31.1553 (5) Å	*T* = 296 K
β = 90.366 (1)°	Prism, brown
*V* = 2851.10 (10) Å^3^	0.08 × 0.06 × 0.05 mm
*Z* = 4	

### Data collection

**Table:**

Bruker SMART APEXII CCD area-detector diffractometer	9270 independent reflections
Radiation source: fine-focus sealed tube	6369 reflections with *I* > 2σ(*I*)
graphite	*R*_int_ = 0.032
φ and ω scans	θ_max_ = 31.5°, θ_min_ = 2.2°
Absorption correction: multi-scan (*SADABS*; Bruker, 2008)	*h* = −13→13
*T*_min_ = 0.622, *T*_max_ = 0.735	*k* = −12→14
34656 measured reflections	*l* = −42→45

### Refinement

**Table:**

Refinement on *F*^2^	Primary atom site location: structure-invariant direct methods
Least-squares matrix: full	Secondary atom site location: difference Fourier map
*R*[*F*^2^ > 2σ(*F*^2^)] = 0.051	Hydrogen site location: inferred from neighbouring sites
*wR*(*F*^2^) = 0.110	H-atom parameters constrained
*S* = 1.03	*w* = 1/[σ^2^(*F*_o_^2^) + (0.0274*P*)^2^ + 21.1246*P*] where *P* = (*F*_o_^2^ + 2*F*_c_^2^)/3
9270 reflections	(Δ/σ)_max_ = 0.001
301 parameters	Δρ_max_ = 3.16 e Å^−^^3^
0 restraints	Δρ_min_ = −2.46 e Å^−^^3^

### Special details

**Table:**

Geometry. Bond distances, angles *etc*. have been calculated using the rounded fractional coordinates. All su's are estimated from the variances of the (full) variance-covariance matrix. The cell e.s.d.'s are taken into account in the estimation of distances, angles and torsion angles
Refinement. Refinement on *F*^2^ for ALL reflections except those flagged by the user for potential systematic errors. Weighted *R*-factors *wR* and all goodnesses of fit *S* are based on *F*^2^, conventional *R*-factors *R* are based on *F*, with *F* set to zero for negative *F*^2^. The observed criterion of *F*^2^ > σ(*F*^2^) is used only for calculating -*R*-factor-obs *etc*. and is not relevant to the choice of reflections for refinement. *R*-factors based on *F*^2^ are statistically about twice as large as those based on *F*, and *R*-factors based on ALL data will be even larger.

### Fractional atomic coordinates and isotropic or equivalent isotropic displacement parameters (Å^2^)

**Table:**

	*x*	*y*	*z*	*U*_iso_*/*U*_eq_	
N1	0.2405 (6)	0.4799 (5)	0.08733 (17)	0.0397 (17)	
N2	0.0962 (7)	0.6082 (6)	0.0433 (2)	0.057 (2)	
N3	0.1215 (6)	0.3771 (6)	0.02881 (19)	0.0470 (17)	
C2	0.1514 (7)	0.4889 (7)	0.0532 (2)	0.0427 (19)	
C4	0.1811 (8)	0.2583 (7)	0.0393 (2)	0.049 (3)	
C5	0.2730 (9)	0.2453 (7)	0.0741 (2)	0.055 (3)	
C6	0.3011 (7)	0.3597 (7)	0.0988 (2)	0.044 (2)	
C7	0.1431 (10)	0.1371 (8)	0.0115 (3)	0.066 (3)	
C8	0.3923 (10)	0.3610 (8)	0.1379 (2)	0.060 (3)	
I1B	0.93595 (6)	0.31957 (5)	0.15828 (2)	0.0591 (2)	
I2B	0.76353 (6)	0.61372 (5)	0.10775 (2)	0.0586 (2)	
I3B	0.53107 (6)	0.23899 (5)	−0.02715 (2)	0.0579 (2)	
O1B	0.7956 (5)	−0.0616 (5)	0.1151 (2)	0.0603 (19)	
O2B	0.9947 (5)	0.0399 (5)	0.09629 (18)	0.0530 (17)	
C9B	0.8596 (7)	0.0346 (6)	0.10190 (18)	0.0340 (17)	
C10B	0.7882 (6)	0.1663 (6)	0.08586 (19)	0.0337 (17)	
C11B	0.8101 (7)	0.2970 (6)	0.1036 (2)	0.0367 (17)	
C12B	0.7461 (7)	0.4111 (6)	0.0840 (2)	0.0400 (19)	
C13B	0.6653 (8)	0.3941 (7)	0.0469 (2)	0.044 (2)	
C14B	0.6441 (7)	0.2637 (7)	0.0302 (2)	0.0407 (19)	
C15B	0.7020 (7)	0.1499 (6)	0.0502 (2)	0.0407 (19)	
I1A	0.50939 (4)	0.95714 (4)	0.15717 (1)	0.0386 (1)	
I2A	0.46466 (6)	1.15333 (7)	0.25657 (2)	0.0728 (2)	
I3A	−0.14705 (5)	1.01436 (7)	0.25537 (2)	0.0637 (2)	
O1A	0.2849 (5)	0.6901 (5)	0.14042 (16)	0.0494 (16)	
O2A	0.1319 (5)	0.8203 (5)	0.10451 (15)	0.0508 (17)	
C9A	0.2086 (6)	0.7931 (6)	0.13696 (19)	0.0357 (17)	
C10A	0.2004 (6)	0.8924 (6)	0.17413 (18)	0.0324 (17)	
C11A	0.3163 (6)	0.9645 (6)	0.19021 (18)	0.0310 (17)	
C12A	0.2980 (7)	1.0461 (7)	0.2266 (2)	0.0387 (17)	
C13A	0.1688 (7)	1.0587 (7)	0.2459 (2)	0.044 (2)	
C14A	0.0549 (7)	0.9898 (7)	0.2285 (2)	0.042 (2)	
C15A	0.0688 (7)	0.9075 (7)	0.19306 (19)	0.0397 (19)	
H1	0.25870	0.55320	0.10200	0.0470*	
H2A	0.04120	0.61530	0.02130	0.0680*	
H2B	0.11480	0.67980	0.05870	0.0680*	
H5	0.31470	0.16060	0.08050	0.0660*	
H7A	0.04290	0.12240	0.01240	0.0990*	
H7B	0.19100	0.05610	0.02190	0.0990*	
H7C	0.17120	0.15540	−0.01750	0.0990*	
H8A	0.43350	0.45110	0.14140	0.0900*	
H8B	0.46600	0.29350	0.13500	0.0900*	
H8C	0.33630	0.33930	0.16260	0.0900*	
H2	1.02910	−0.03670	0.10070	0.0790*	
H13B	0.62560	0.47070	0.03330	0.0530*	
H15B	0.68310	0.06170	0.03970	0.0480*	
H13A	0.15830	1.11290	0.27030	0.0530*	
H15A	−0.00950	0.86190	0.18170	0.0480*	

### Atomic displacement parameters (Å^2^)

**Table:**

	*U*^11^	*U*^22^	*U*^33^	*U*^12^	*U*^13^	*U*^23^
N1	0.044 (3)	0.032 (3)	0.043 (3)	−0.001 (2)	−0.003 (2)	−0.012 (2)
N2	0.066 (4)	0.045 (3)	0.060 (4)	0.013 (3)	−0.026 (3)	−0.022 (3)
N3	0.052 (3)	0.040 (3)	0.049 (3)	−0.006 (3)	−0.001 (3)	−0.017 (3)
C2	0.046 (4)	0.039 (3)	0.043 (3)	−0.004 (3)	−0.001 (3)	−0.014 (3)
C4	0.061 (5)	0.043 (4)	0.043 (4)	−0.006 (3)	0.010 (3)	−0.012 (3)
C5	0.077 (5)	0.029 (3)	0.060 (5)	0.006 (3)	0.004 (4)	−0.009 (3)
C6	0.049 (4)	0.039 (3)	0.044 (4)	0.002 (3)	0.002 (3)	−0.004 (3)
C7	0.098 (7)	0.046 (4)	0.055 (5)	−0.018 (4)	0.008 (4)	−0.025 (4)
C8	0.082 (6)	0.048 (4)	0.049 (4)	0.010 (4)	−0.011 (4)	−0.003 (3)
I1B	0.0803 (4)	0.0520 (3)	0.0447 (3)	0.0091 (3)	−0.0230 (2)	−0.0098 (2)
I2B	0.0829 (4)	0.0309 (2)	0.0617 (3)	0.0075 (2)	−0.0179 (3)	−0.0117 (2)
I3B	0.0672 (3)	0.0557 (3)	0.0505 (3)	0.0017 (2)	−0.0239 (2)	−0.0032 (2)
O1B	0.048 (3)	0.034 (3)	0.099 (4)	0.003 (2)	0.008 (3)	0.014 (3)
O2B	0.046 (3)	0.041 (3)	0.072 (3)	0.009 (2)	−0.004 (2)	0.013 (2)
C9B	0.044 (3)	0.027 (3)	0.031 (3)	0.003 (2)	0.000 (2)	−0.005 (2)
C10B	0.035 (3)	0.031 (3)	0.035 (3)	0.002 (2)	0.001 (2)	−0.001 (2)
C11B	0.038 (3)	0.034 (3)	0.038 (3)	0.003 (2)	−0.001 (2)	−0.007 (2)
C12B	0.051 (4)	0.029 (3)	0.040 (3)	0.000 (3)	0.001 (3)	−0.005 (2)
C13B	0.057 (4)	0.030 (3)	0.045 (4)	0.010 (3)	−0.010 (3)	0.000 (3)
C14B	0.047 (4)	0.037 (3)	0.038 (3)	0.005 (3)	−0.011 (3)	−0.007 (3)
C15B	0.048 (4)	0.032 (3)	0.042 (3)	0.006 (3)	−0.005 (3)	−0.009 (3)
I1A	0.0379 (2)	0.0348 (2)	0.0432 (2)	0.0043 (2)	0.0009 (2)	−0.0010 (2)
I2A	0.0538 (3)	0.0821 (4)	0.0824 (4)	−0.0050 (3)	−0.0137 (3)	−0.0484 (3)
I3A	0.0448 (3)	0.0929 (4)	0.0535 (3)	0.0138 (3)	0.0113 (2)	0.0087 (3)
O1A	0.059 (3)	0.034 (2)	0.055 (3)	0.014 (2)	−0.017 (2)	−0.013 (2)
O2A	0.064 (3)	0.044 (3)	0.044 (3)	0.017 (2)	−0.022 (2)	−0.017 (2)
C9A	0.036 (3)	0.032 (3)	0.039 (3)	0.000 (2)	−0.005 (2)	−0.009 (2)
C10A	0.037 (3)	0.025 (3)	0.035 (3)	0.003 (2)	−0.007 (2)	−0.002 (2)
C11A	0.035 (3)	0.028 (3)	0.030 (3)	0.006 (2)	0.000 (2)	−0.002 (2)
C12A	0.041 (3)	0.037 (3)	0.038 (3)	0.006 (3)	−0.005 (3)	−0.010 (3)
C13A	0.053 (4)	0.045 (4)	0.035 (3)	0.011 (3)	−0.004 (3)	−0.012 (3)
C14A	0.045 (4)	0.049 (4)	0.033 (3)	0.010 (3)	0.003 (3)	0.005 (3)
C15A	0.043 (4)	0.041 (3)	0.035 (3)	−0.001 (3)	−0.003 (3)	−0.001 (3)

### Geometric parameters (Å, °)

**Table:**

I1B—C11B	2.084 (6)	C7—H7A	0.9600
I2B—C12B	2.100 (6)	C7—H7B	0.9600
I3B—C14B	2.090 (6)	C7—H7C	0.9600
I1A—C11A	2.105 (6)	C8—H8C	0.9600
I2A—C12A	2.102 (7)	C8—H8A	0.9600
I3A—C14A	2.105 (7)	C8—H8B	0.9600
O1B—C9B	1.185 (8)	C9B—C10B	1.524 (8)
O2B—C9B	1.293 (8)	C10B—C11B	1.394 (8)
O2B—H2	0.8200	C10B—C15B	1.383 (9)
O1A—C9A	1.235 (7)	C11B—C12B	1.397 (9)
O2A—C9A	1.268 (7)	C12B—C13B	1.392 (9)
N1—C2	1.356 (8)	C13B—C14B	1.378 (9)
N1—C6	1.343 (8)	C14B—C15B	1.377 (9)
N2—C2	1.303 (9)	C13B—H13B	0.9300
N3—C2	1.350 (9)	C15B—H15B	0.9300
N3—C4	1.320 (9)	C9A—C10A	1.507 (8)
N1—H1	0.8600	C10A—C11A	1.391 (8)
N2—H2A	0.8600	C10A—C15A	1.389 (9)
N2—H2B	0.8600	C11A—C12A	1.393 (9)
C4—C5	1.391 (10)	C12A—C13A	1.372 (9)
C4—C7	1.500 (11)	C13A—C14A	1.376 (9)
C5—C6	1.373 (9)	C14A—C15A	1.368 (9)
C6—C8	1.489 (10)	C13A—H13A	0.9300
C5—H5	0.9300	C15A—H15A	0.9300
			
I1A···O1A	3.382 (5)	N3···N2^x^	3.042 (9)
I1A···O1B^i^	3.023 (5)	N3···H2A^x^	2.1900
I1A···C9B^i^	3.819 (6)	C4···O2B^vi^	3.281 (9)
I1A···I2A	3.6584 (8)	C5···O2B^vi^	3.374 (9)
I1A···I2A^ii^	3.9877 (8)	C7···O2B^vi^	3.144 (11)
I1B···I2B	3.6327 (7)	C8···O1A	3.341 (9)
I1B···I3A^ii^	3.8352 (8)	C9A···N1	3.414 (8)
I1B···O2B	3.371 (5)	C9A···O2B^viii^	3.371 (8)
I2A···I1A	3.6584 (8)	C9B···I1A^vii^	3.819 (6)
I2A···I3A^iii^	3.9136 (8)	C9B···O2A^ix^	3.308 (8)
I2A···I1A^iv^	3.9878 (7)	C10A···O2B^viii^	3.413 (8)
I2B···I3B^v^	4.0025 (8)	C15A···O2B^viii^	3.346 (8)
I2B···I1B	3.6327 (7)	C4···H2A^x^	3.0700
I2B···O1B^i^	3.162 (5)	C7···H15B^xi^	3.0000
I3A···I2A^vi^	3.9136 (8)	C9A···H2^viii^	2.6200
I3A···I1B^iv^	3.8351 (8)	C9A···H2B	2.8100
I3B···I2B^v^	4.0026 (8)	C9A···H1	2.6100
I1A···H8B^i^	3.3500	C10A···H2^viii^	2.8800
I3B···H13B^v^	3.1800	C15A···H2^viii^	2.9500
O1A···C8	3.341 (9)	H1···O1A	1.8000
O1A···I1A	3.382 (5)	H1···O2A	2.8500
O1A···N1	2.652 (7)	H1···H8A	2.2800
O1B···I1A^vii^	3.023 (5)	H1···C9A	2.6100
O1B···I2B^vii^	3.162 (5)	H1···H2B	2.2700
O2A···O2B^viii^	2.501 (7)	H2···O2A^ix^	1.6900
O2A···C9B^viii^	3.308 (8)	H2···C15A^ix^	2.9500
O2A···N2	2.819 (8)	H2···C9A^ix^	2.6200
O2B···C4^iii^	3.281 (9)	H2···C10A^ix^	2.8800
O2B···C15A^ix^	3.346 (8)	H2A···N3^x^	2.1900
O2B···O2A^ix^	2.501 (7)	H2A···C4^x^	3.0700
O2B···C10A^ix^	3.413 (8)	H2B···O2A	1.9800
O2B···I1B	3.371 (5)	H2B···C9A	2.8100
O2B···C7^iii^	3.144 (11)	H2B···H1	2.2700
O2B···C5^iii^	3.374 (9)	H5···H8B	2.5600
O2B···C9A^ix^	3.371 (8)	H5···H7B	2.3900
O1A···H1	1.8000	H7A···O2B^vi^	2.7700
O1A···H8A	2.7100	H7B···H5	2.3900
O1B···H15A^ix^	2.8600	H7B···H15B^xi^	2.5400
O1B···H15B	2.8400	H8A···H1	2.2800
O2A···H2B	1.9800	H8A···O1A	2.7100
O2A···H2^viii^	1.6900	H8B···I1A^vii^	3.3500
O2A···H15A	2.7900	H8B···H5	2.5600
O2A···H1	2.8500	H13B···I3B^v^	3.1800
O2B···H7A^iii^	2.7700	H15A···O1B^viii^	2.8600
N1···C9A	3.414 (8)	H15A···O2A	2.7900
N1···O1A	2.652 (7)	H15B···O1B	2.8400
N2···N3^x^	3.042 (9)	H15B···C7^xi^	3.0000
N2···O2A	2.819 (8)	H15B···H7B^xi^	2.5400
			
C9B—O2B—H2	109.00	C9B—C10B—C11B	124.2 (5)
C2—N1—C6	121.8 (5)	I1B—C11B—C10B	120.1 (4)
C2—N3—C4	118.0 (6)	C10B—C11B—C12B	118.7 (6)
C6—N1—H1	119.00	I1B—C11B—C12B	121.2 (4)
C2—N1—H1	119.00	I2B—C12B—C11B	123.3 (5)
C2—N2—H2A	120.00	C11B—C12B—C13B	120.3 (6)
H2A—N2—H2B	120.00	I2B—C12B—C13B	116.4 (5)
C2—N2—H2B	120.00	C12B—C13B—C14B	120.0 (6)
N2—C2—N3	119.6 (6)	I3B—C14B—C13B	120.0 (5)
N1—C2—N3	121.1 (6)	C13B—C14B—C15B	120.3 (6)
N1—C2—N2	119.3 (6)	I3B—C14B—C15B	119.7 (5)
C5—C4—C7	121.7 (6)	C10B—C15B—C14B	120.2 (6)
N3—C4—C5	122.4 (6)	C12B—C13B—H13B	120.00
N3—C4—C7	115.9 (6)	C14B—C13B—H13B	120.00
C4—C5—C6	118.8 (6)	C10B—C15B—H15B	120.00
C5—C6—C8	125.1 (6)	C14B—C15B—H15B	120.00
N1—C6—C5	117.9 (6)	O1A—C9A—O2A	124.6 (6)
N1—C6—C8	117.1 (6)	O1A—C9A—C10A	118.7 (5)
C6—C5—H5	121.00	O2A—C9A—C10A	116.7 (5)
C4—C5—H5	121.00	C9A—C10A—C11A	123.5 (5)
C4—C7—H7B	109.00	C9A—C10A—C15A	116.4 (5)
C4—C7—H7C	109.00	C11A—C10A—C15A	120.1 (5)
H7A—C7—H7B	110.00	I1A—C11A—C10A	119.5 (4)
H7A—C7—H7C	110.00	I1A—C11A—C12A	122.0 (4)
H7B—C7—H7C	110.00	C10A—C11A—C12A	118.4 (5)
C4—C7—H7A	109.00	I2A—C12A—C11A	123.0 (5)
C6—C8—H8A	109.00	I2A—C12A—C13A	115.5 (5)
C6—C8—H8C	109.00	C11A—C12A—C13A	121.5 (6)
H8A—C8—H8B	109.00	C12A—C13A—C14A	118.9 (6)
H8A—C8—H8C	109.00	I3A—C14A—C13A	120.0 (5)
H8B—C8—H8C	109.00	I3A—C14A—C15A	118.6 (5)
C6—C8—H8B	110.00	C13A—C14A—C15A	121.4 (6)
O1B—C9B—O2B	125.9 (6)	C10A—C15A—C14A	119.7 (6)
O1B—C9B—C10B	122.9 (6)	C12A—C13A—H13A	121.00
O2B—C9B—C10B	111.1 (5)	C14A—C13A—H13A	121.00
C9B—C10B—C15B	115.2 (5)	C10A—C15A—H15A	120.00
C11B—C10B—C15B	120.5 (5)	C14A—C15A—H15A	120.00
			
C6—N1—C2—N2	−179.6 (6)	I2B—C12B—C13B—C14B	−177.5 (5)
C6—N1—C2—N3	−1.0 (10)	C11B—C12B—C13B—C14B	2.3 (10)
C2—N1—C6—C5	1.7 (10)	C12B—C13B—C14B—I3B	−177.0 (5)
C2—N1—C6—C8	−177.5 (6)	C12B—C13B—C14B—C15B	0.3 (10)
C4—N3—C2—N1	0.3 (10)	I3B—C14B—C15B—C10B	173.8 (5)
C2—N3—C4—C7	179.3 (6)	C13B—C14B—C15B—C10B	−3.5 (10)
C4—N3—C2—N2	178.9 (6)	O1A—C9A—C10A—C11A	61.3 (8)
C2—N3—C4—C5	−0.3 (10)	O1A—C9A—C10A—C15A	−117.3 (6)
N3—C4—C5—C6	1.0 (11)	O2A—C9A—C10A—C11A	−120.8 (6)
C7—C4—C5—C6	−178.7 (7)	O2A—C9A—C10A—C15A	60.6 (7)
C4—C5—C6—C8	177.5 (7)	C9A—C10A—C11A—I1A	8.1 (8)
C4—C5—C6—N1	−1.6 (10)	C9A—C10A—C11A—C12A	−175.6 (6)
O1B—C9B—C10B—C11B	119.6 (7)	C15A—C10A—C11A—I1A	−173.4 (5)
O1B—C9B—C10B—C15B	−63.2 (8)	C15A—C10A—C11A—C12A	2.9 (9)
O2B—C9B—C10B—C11B	−64.7 (8)	C9A—C10A—C15A—C14A	176.3 (6)
O2B—C9B—C10B—C15B	112.4 (6)	C11A—C10A—C15A—C14A	−2.3 (9)
C9B—C10B—C11B—I1B	−4.6 (8)	I1A—C11A—C12A—I2A	−6.9 (7)
C9B—C10B—C11B—C12B	175.4 (6)	I1A—C11A—C12A—C13A	174.8 (5)
C15B—C10B—C11B—I1B	178.4 (5)	C10A—C11A—C12A—I2A	176.9 (4)
C15B—C10B—C11B—C12B	−1.6 (9)	C10A—C11A—C12A—C13A	−1.5 (9)
C9B—C10B—C15B—C14B	−173.1 (6)	I2A—C12A—C13A—C14A	−179.1 (5)
C11B—C10B—C15B—C14B	4.2 (9)	C11A—C12A—C13A—C14A	−0.6 (10)
I1B—C11B—C12B—I2B	−1.9 (8)	C12A—C13A—C14A—I3A	−177.0 (5)
I1B—C11B—C12B—C13B	178.4 (5)	C12A—C13A—C14A—C15A	1.3 (10)
C10B—C11B—C12B—I2B	178.1 (5)	I3A—C14A—C15A—C10A	178.4 (5)
C10B—C11B—C12B—C13B	−1.6 (10)	C13A—C14A—C15A—C10A	0.2 (10)

Symmetry codes: (i) *x*, *y*+1, *z*; (ii) −*x*+1, *y*−1/2, −*z*+1/2; (iii) *x*+1, *y*, *z*; (iv) −*x*+1, *y*+1/2, −*z*+1/2; (v) −*x*+1, −*y*+1, −*z*; (vi) *x*−1, *y*, *z*; (vii) *x*, *y*−1, *z*; (viii) *x*−1, *y*+1, *z*; (ix) *x*+1, *y*−1, *z*; (x) −*x*, −*y*+1, −*z*; (xi) −*x*+1, −*y*, −*z*.

### Hydrogen-bond geometry (Å, °)

**Table:**

*D*—H···*A*	*D*—H	H···*A*	*D*···*A*	*D*—H···*A*
N1—H1···O1A	0.86	1.80	2.652 (7)	170.
N2—H2B···O2A	0.86	1.98	2.819 (8)	166.
N2—H2A···N3^x^	0.86	2.19	3.042 (9)	172.
O2B—H2···O2A^ix^	0.82	1.69	2.501 (7)	167.

Symmetry codes: (x) −*x*, −*y*+1, −*z*; (ix) *x*+1, *y*−1, *z*.
